# Inhibitors of p53 Apoptosis‐Stimulating Protein Mitigate Acute Kidney Injury by Modulating the HIF‐1α/SLC7A11 Pathway to Suppress Ferroptosis

**DOI:** 10.1111/jcmm.70580

**Published:** 2025-06-10

**Authors:** Peng Kang, Xiangjun Zhou, Sheng Zhao, Weimin Yu, Zehua Ye, Fan Cheng

**Affiliations:** ^1^ Department of Urology Renmin Hospital of Wuhan University Wuhan China

**Keywords:** AKI, ferroptosis, hypoxia‐inducible factor 1α, iASPP, ischaemia–reperfusion

## Abstract

Acute kidney injury (AKI) is a complex disease caused by different causes, especially ischaemia–reperfusion (I/R) injury. Ferroptosis is the main form of I/R‐induced organ injury, and blocking ferroptosis has demonstrated therapeutic potential in ameliorating organ injury. We investigated the roles of apoptosis‐stimulating protein of p53 (iASPP) and hypoxia‐inducible factor‐1α (HIF‐1α) in ferroptosis during renal I/R injury. HIF‐1α gene was knocked out in a hypoxia/reoxygenation model of renal tubular epithelial cells, and iASPP overexpression and knockdown plasmids were transfected. In I/R mouse models, conditional knockout of HIF‐1α mice and injection of overexpressed iASPP adeno‐associated viruses were used to validate downstream ferroptosis‐related changes. The results showed that the ferroptosis level of mice in the I/R group was increased, and the addition of Ferrostatin‐1 (Fer‐1) and FG‐4592 could alleviate the ferroptosis. HIF‐1α conditional knockout mice showed exacerbated ferroptosis. HIF‐1α can directly interact with SLC7A11, a key ferroptosis regulator, modulating ferroptosis progression. Similar to HIF‐1α, iASPP expression was significantly increased in the I/R group, and overexpression of iASPP upregulated HIF‐1α and SLC7A11 expression, consequently mitigating ferroptosis‐mediated damage. In summary, our study suggests that iASPP exerts renal protection during I/R injury by regulating the HIF‐1α/SLC7A11 axis to suppress ferroptosis.

## 1 Background

Acute kidney injury (AKI) is a major global health problem, resulting in high morbidity and mortality. It is characterised by a rapid decline in renal function, accumulation of metabolic wastes and disturbances in body fluids, electrolytes and acid–base balance [1, 2, 3]. Despite 30%–70% of AKI cases progressing to chronic kidney disease (CKD) or end‐stage renal disease (ESRD), current therapeutic options remain limited to dialysis with inadequate efficacy in reducing mortality, mitigating damage or accelerating recovery. Therefore, elucidating the precise mechanisms underlying AKI pathogenesis holds significant clinical value.

Dixon et al. first defined ferroptosis in 2012 as an oxidative damage‐driven, non‐apoptotic form of cell death [4]. This process is initiated by intracellular iron overload, which promotes lipid peroxidation and subsequent cell membrane destruction [5]. Glutathione peroxidase 4 (GPX4) serves as a crucial enzyme that converts toxic lipid peroxides into harmless lipids via glutathione metabolism, thereby suppressing ferroptosis [6]. Cystine glutamate reverse transporter (xCT/SLC7A11) regulates glutathione synthesis and GPX4 activity by mediating cystine uptake. Inhibition of xCT activity impairs GPX4 antioxidant capacity, leading to lipid peroxide accumulation and enhanced cellular susceptibility to ferroptosis [7, 8]. Studies on I/R damaged mouse models, GPX4 knockout mice and folate‐induced AKI have shown that ferroptosis inhibitors reduce renal tubular cell death and acute kidney failure [9, 10].

Ischaemia–reperfusion (I/R) injury, occurring when blood flow is restored following prolonged ischaemia, constitutes a major aetiology to AKI in hypotensive or septic patients [11, 12]. Hypoxia‐inducible factor (HIF‐1α) upregulation has been identified as a hallmark of I/R injury, particularly in hypoxic renal tubules [13]. While HIF‐1α activation functions as a key mediator of hypoxic stress responses and confers renal protection, its dysregulated activation in renal tubular cells may paradoxically exacerbate kidney disease progression [14, 15]. Although HIF‐1α plays significant roles in renal pathophysiology, its precise mechanistic involvement requires further elucidation.

It is well known that p53 is closely related to the occurrence of ferroptosis. Through downregulation of SLC7A11, p53 inhibits cystine uptake and impairs GPX4 function, thereby promoting reactive oxygen species (ROS) accumulation and ferroptosis induction [16]. ASPP1, ASPP2 and inhibitor of apoptosis‐stimulating protein of p53 (iASPP), as members of the ASPP (apoptosis‐stimulating protein of p53) family, combine ASPP1 and ASPP2 with p53 to promote apoptosis, while iASPP antagonises this process. Specifically, ASPP1 binding facilitates p53 nuclear translocation, exacerbating I/R injury through enhanced apoptotic signalling. Targeting ASPP1‐p53 interactions or their nuclear transport mechanisms represents a promising therapeutic strategy for cardiac I/R injury [17]. Li et al. found that iASPP could alleviate acute lung injury induced by intestinal I/R through ferroptosis dependent on Nrf2 inhibitory cells [18]. In addition, other studies have found that iASPP can regulate HIF‐1α transcriptional activity and downstream target genes expression in an HIF‐1α‐dependent manner, significantly enhancing angiogenesis and glycolysis [19]. Therefore, we hypothesise that iASPP may regulate ferroptosis during renal ischaemia through HIF‐1α‐mediated pathways. Our renal I/R experiments revealed coordinated expression changes in iASPP and HIF‐1α, with subsequent overexpression and knockout studies confirming their regulatory effects on HIF‐1α and downstream ferroptosis‐related biomarkers.

In summary, these findings indicate the potential therapeutic targeting of iASPP and HIF‐1α in ferroptosis‐associated AKI. To systematically investigate their mechanistic roles in ferroptosis modulation and evaluate translational applications, we designed this study.

## 2 Materials and Methods

### 2.1 Animal Model of I/R‐Induced AKI

All experiments were performed under the approval of the Animal Ethics Committee of the Renmin Hospital of Wuhan University (approval number: WDRM20200904) and in accordance with the principles of the relevant guidelines. Male C57BL/6 mice aged 6–8 weeks (from Suzhou Saiye Biologicals) were randomly divided into different groups (*n* = 6 per group). All mice were fasted for 24 h before surgery with unlimited water access. For pharmacological interventions, the I/*R* + Fer‐1 group received 1.5 mg/kg Ferrostatin‐1 (MedChemExpress, HY‐100579) via tail vein injection 30 min pre‐ischaemia, while the I/*R* + FG‐4592 group was administered 50 mg/kg FG‐4592 (MedChemExpress, HY‐13426) through the same route using identical timing. Surgical procedures included intraperitoneal injection of 3% sodium pentobarbital (50 mg/kg) followed by temperature maintenance at 36.5°C using a thermostatic pad. Bilateral renal pedicle exposure was achieved through flank incisions, with ischaemia induced by non‐invasive vascular clamps for 30 min. Renal artery occlusion was confirmed by loss of renal pallor within 2 min of clamping. The clamps were removed to allow reperfusion at specified timepoints, followed by surgical wound closure. Sham‐operated control animals underwent identical anaesthesia and surgical exposure without renal artery clamping. Blood and renal tissues were collected at designated reperfusion intervals (0, 4, 8 and 24 h) for subsequent analyses. Prior to the renal artery occlusion, the I/*R* + Ferrostatin‐1 group received an injection of 1.5 mg/kg Fer‐1 through the tail vein. Postoperative mice were maintained in a temperature‐controlled environment (24°C–30°C) with ad libitum access to food and water. To generate renal‐specific HIF‐1α knockout mice, HIF‐1α^flox/flox^ mice were bred with Ggt1‐Cre transgenic mice. All adult mice were housed under specific pathogen‐free conditions.

### 2.2 Adenovirus‐Mediated iASPP Expression in Mice

The iASPP‐overexpressing adenovirus was obtained from Genechem (Shanghai, China). Under 2% isoflurane anaesthesia, a mixture containing 10 μg iASPP plasmid and 2 μL Polyplus transfection reagent in 600 μL 10% glucose solution was retrograde‐infused into the left renal artery. Transient renal arteriovenous occlusion (5 min) was applied to enhance localised delivery efficiency. After the injection, Rich‐Mar's Sonitron 2000 ultrasonic transducer was positioned over the renal parenchyma. Ultrasound parameters were set to 1 MHz frequency with 10% output power, delivering 60‐s pulses at 30‐s intervals. Subsequently, the renal arteriovenous obstruction was removed and the renal blood flow was restored to normal.

This protocol achieved efficient renal DNA delivery without nephrotoxicity. iASPP expression was validated through immunohistochemical localisation, western blot quantification and qPCR analysis of renal tissues.

### 2.3 Cell Culture

HK2 cells were obtained from Genomeditech Biotechnology Co. Ltd. (Shanghai, China) and cultured in DMEM/F12 (Gibco, Hangzhou, China) with 10% fetal bovine serum (Gibco, NY, USA), 100 U/mL penicillin and 100 μg/mL streptomycin (YEASEN Biotech Co Ltd., Shanghai, China) at 37°C and 5% CO_2_. Cells at log‐phase growth were seeded into 6‐well plates. Hypoxic conditions were established using a tri‐gas incubator (5% CO_2_, 94% N_2_ and 1% O_2_) at 37°C for 24 h, followed by reoxygenation for 0, 4, 8 and 12 h. Post‐treatment, cells were returned to normoxic conditions (37°C) for 2 h.

### 2.4 Renal Function and Histopathological Analysis

Mice kidney function was evaluated by analysing serum creatinine levels with the FUJIDRI‐CHEM 7000i biochemical analyser from Fujifilm Corporation. Kidney tissues were fixed in 4% paraformaldehyde, then embedded in paraffin and cut into 4‐μm‐thick sections. The sections were stained with Haematoxylin–eosin and examined using a Leica DM4000 microscope. Evaluation of renal tubular epithelium damage included assessing loss of tubular brush border, dilation and destruction of the tubular epithelium. Renal tubular injury was semi‐quantitatively scored based on the percentage of damaged tubules: 0 for no injury, 1 for < 25%, 2 for 25%–50%, 3 for 50%–75% and 4 for > 75%. Two pathologists, unaware of the experiments, conducted the assessment.

### 2.5 Western Blot Analysis

Renal tissues were homogenised with beads in RIPA lysis buffer (Beyotime, Shanghai, China), followed by centrifugation (12,000 *g*, 15 min, 4°C) to collect supernatants. Protein concentrations were determined via BCA assay (Thermo Fisher Scientific, Waltham, MA, USA). Lysates were heat‐denatured (95°C, 5 min), resolved by SDS‐PAGE and transferred to PVDF membranes. After blocking with 5% non‐fat milk, membranes were incubated overnight at 4°C with primary antibodies. Following TBST washes, membranes were incubated with HRP‐conjugated secondary antibodies (1:5000; ABclonal) for 1 h at room temperature. Protein bands were visualised using ECL substrate (Millipore, Burlington, MA, USA) and quantified with ImageJ software.

The main antibodies used in Western blots were anti‐iASPP (1:1000, Abcam, Cambridge, MA, USA), anti‐HIF‐1α (1:1000, Abcam), anti‐GPX4 (1:500, ABclonal, Wuhan, CHINA), anti‐SLC7A11/xCT (1:500, ABclonal), anti‐ACSL4 (1:10,000, ABclonal), anti‐KIM‐1 (1:2000, ABclonal), anti‐NGAL (1:2000, ABclonal), anti‐FTH1 (1:500, ABclonal) and anti‐GAPDH (1:10,000, ABclonal). The trials were performed three times, and the data from Western blot tests were assessed with Image J software from the National Institutes of Health in Bethesda, Maryland, USA.

### 2.6 RT‐PCR Analysis

Following kidney harvest, tissues were immediately flash‐frozen in liquid nitrogen, pulverised and processed with TRIzol reagent (Invitrogen, Carlsbad, CA, USA). Total RNA was isolated through sequential chloroform extraction, isopropanol precipitation and 75% ethanol washes, followed by resuspension in RNase‐free water. RNA purity and concentration were determined spectrophotometrically (NanoDrop, Thermo Fisher Scientific, Waltham, MA, USA), with aliquots stored at −80°C.

For reverse transcription, 3 μg of total RNA was converted to cDNA using a High‐Capacity cDNA Reverse Transcription Kit (Applied Biosystems, Foster City, CA, USA). Quantitative PCR amplification was performed for 40 cycles: 95°C for 10 s (denaturation), 65°C for 30 s (annealing/extension). Relative gene expression was calculated via the $2^{-\Delta \Delta C_{T}}$ method. Primer sequences and PCR settings are as follows: SLC7A11: forward, 5ʹ‐ATGCAGTGGCAGTGACCTTT‐3ʹ, reverse, 5ʹ‐GGCAACAAAGATCGGAACTG‐3ʹ; HIF‐1α: forward, 5ʹ‐ACCTTCATCGGAAACTCCAAAG‐3ʹ, reverse, 5ʹ‐CTGTTAGGCTGGGAAAAGTTAGG‐3ʹ; iASPP: forward, 5ʹ‐GAAATCACTGGGGACAGGAA‐3ʹ, reverse, 5ʹ‐CCCAGGAATATCCAGTGGTG‐3ʹ; GPX4: forward, 5ʹ‐AGAGATCAAAGAGTTCGCCGC‐3ʹ, reverse, 5ʹ‐TCTTCATCCACTTCCACAGCG‐3ʹ.

### 2.7 Immunohistochemical Analysis

Fixed kidney specimens were dehydrated through a graded ethanol series, paraffin‐embedded and sectioned (4 μm) using a rotary microtome (Leica, Wetzlar, Germany). After deparaffinisation in xylene and rehydration, antigen retrieval was performed in preheated 0.01 M citrate buffer (pH 6.0) at 95°C for 5 min, followed by cooling to room temperature. Afterward, the segments were washed three times with PBS, then exposed to a 3% hydrogen peroxide solution and placed in an incubator at 37°C for 50 min. Following this, primary antibody against iASPP (1:150, Abcam, Cambridge, MA, USA) was applied, and the sections were left to incubate at 4°C overnight. After PBS washes, HRP‐conjugated secondary antibody incubation (37°C, 1 h) was performed. Diaminobenzidine (DAB; Sigma‐Aldrich, St. Louis, MO, USA) chromogenic development was monitored microscopically, followed by DAPI counterstaining (Thermo Fisher Scientific) and mounting with Permount (Fisher Scientific, Hampton, NH, USA). Quantitative analysis was conducted using ImageJ software.

### 2.8 Immunofluorescence Staining

HK2 cells grown on glass coverslips were fixed with 4% paraformaldehyde for 15 min at room temperature. After permeabilisation with 0.1% Triton X‐100, samples were blocked with 5% BSA and incubated with primary antibodies at 4°C overnight. After washing the slides three times with PBST, they were then incubated with a fluorescent secondary antibody at room temperature for 1 h. Nuclei were counterstained with DAPI (1 μg/mL; Sigma‐Aldrich), and slides were mounted with ProLong Diamond Antifade Mountant (Thermo Fisher Scientific). Images were acquired using a fluorescence microscope (Olympus IX83, Tokyo, Japan) and analysed with Image‐Pro Plus software. The main antibodies used in immunofluorescence staining were anti‐GPX4 (1:50, ABclonal, Wuhan, CHINA), anti‐SLC7A11/xCT (1:50, ABclonal) and anti‐HIF‐1α (1:100, Abcam).

### 2.9 TUNEL Assay

Renal tissues and HK2 cells were analysed using a fluorescent TUNEL assay kit, following the provided guidelines. Frozen kidney sections were fixed in 4% paraformaldehyde, paraffin‐embedded, sectioned (5 μm), dewaxed in xylene and rehydrated through a graded ethanol series prior to analysis. HK2 cells grown on coverslips were fixed with 4% PFA for 15 min at room temperature. TUNEL‐positive nuclei were quantified by capturing 10 random fields per sample using an Olympus FV3000 confocal microscope (Tokyo, Japan) under double‐blind conditions.

### 2.10 Malondialdehyde (MDA) Assay

The concentration of malondialdehyde in renal tissue was assessed using the thiobarbituric acid reactive substances method. The tissue homogenate was mixed with the MDA working solution according to the manufacturer's guidelines, then heated at 100°C for 15 min. The spectrophotometer was used to measure absorbance at 532 nm, and the MDA concentration in kidney tissue was calculated using a standard curve (nmol/L).

### 2.11 Quantification of Glutathione (GSH)

The cells were treated as instructed, and the GSH‐GSH/GSSG ratio test kit (Abcam, ab138881) was utilised to evaluate cellular GSH levels. Briefly, cells were lysed and centrifuged (12,000 *g*, 10 min, 4°C) to collect supernatants. Total GSH working solution (150 μL) was added to 96‐well plates containing samples/standards, followed by NADPH solution (50 μL) incubation. After 20‐min incubation at 25°C, absorbance at 412 nm was measured. The determination of GSH content within the sample was subsequently achieved through the utilisation of the standard curve in conjunction with the absorbance value of each individual sample.

### 2.12 Iron Measurement

Mouse renal tissue was harvested and iron concentrations were determined with an iron assay kit following the guidelines provided by the manufacturer. Fresh kidney tissue was mixed with PBS and centrifuged to obtain the supernatant, which was then used to measure the iron levels using an iron content detection kit.

### 2.13 Detection of Reactive Oxygen Species (ROS) by Flow Cytometry

HK2 cells were seeded in six‐well plates with a concentration of 1 × 10^6^ cells per well. Various groups of cells were harvested, including a blank control group, and the plates were maintained in a 37°C, 5% CO_2_ cell culture incubator for 72 h. After incubation, the medium was taken out, the cells were rinsed with PBS twice and a fluorescent dye, DCFH‐DA, was introduced at a concentration of 10 μmol/L. The cells continued to be cultured in a 37°C, 5% CO_2_ cell culture incubator for 30 min and then resuspended in 500 μL PBS for detection by flow cytometry. Incubate for 30 min at 37°C in a 5% CO_2_ cell culture incubator, wash twice with pre‐cooled PBS, resuspend cells in 500 μL PBS and analyse using flow cytometry. Analysis was conducted with Flowjo V10 software from Flowjo LLC in the United States. The voltage, acquisition rate and gating settings remained consistent across all cell groups during the flow assay.

### 2.14 Chromatin Immunoprecipitation (ChIP)

Forty‐eight hours post‐transfection of HK2 cells, cellular samples were obtained for cross‐linking ChIP utilising a chromatin immunoprecipitation kit. qPCR amplification used SLC7A11‐specific primers: Forward: 5ʹ‐TTTGAGCATGGAGGGTTGAAG‐3ʹ; Reverse: 5ʹ‐GATGGAGGAGAGGTGATATTGAGAA‐3ʹ. Results were normalised to input DNA and expressed as % input.

### 2.15 Ultrastructure Observation by Transmission Electron Microscope

Renal cortical fragments (1 mm^3^) were fixed in 2.5% glutaraldehyde/0.1 M phosphate buffer (pH 7.4) for 24 h at 4°C. After PBS washes, post‐fixation with 1% osmium tetroxide was performed for 2 h. Dehydration through an ethanol series (50%–100%) and acetone infiltration preceded embedding in EPON 812 resin (Electron Microscopy Sciences, Hatfield, PA, USA). Ultrathin sections (70 nm) were stained with 2% uranyl acetate and Reynolds' lead citrate. Mitochondrial ultrastructure was analysed using a Hitachi HT7800 TEM (Tokyo, Japan) at 8000× initial magnification.

### 2.16 Statistical Analysis

Statistical analysis was performed using SPSS 26.0 for data management and analysis, and GraphPad Prism 5 software for statistical calculations and graphical representation. Measurement data were presented as mean ± standard deviation ($\bar{x}$ ± s). For comparisons between two groups, the independent samples *t*‐test was employed. When comparing three or more groups, one‐way analysis of variance (ANOVA) was first conducted to assess overall differences. For post hoc analysis following significant ANOVA results (*p* < 0.05), the Least Significant Difference (LSD) test was used for pairwise comparisons. Multiple comparison adjustments were not applied given the exploratory nature of the LSD method. The statistical significance threshold was maintained at *p* < 0.05 for all analyses.

## 3 Results

### 3.1 I/R‐Induced AKI Enhances Ferroptosis

**FIGURE 1:**
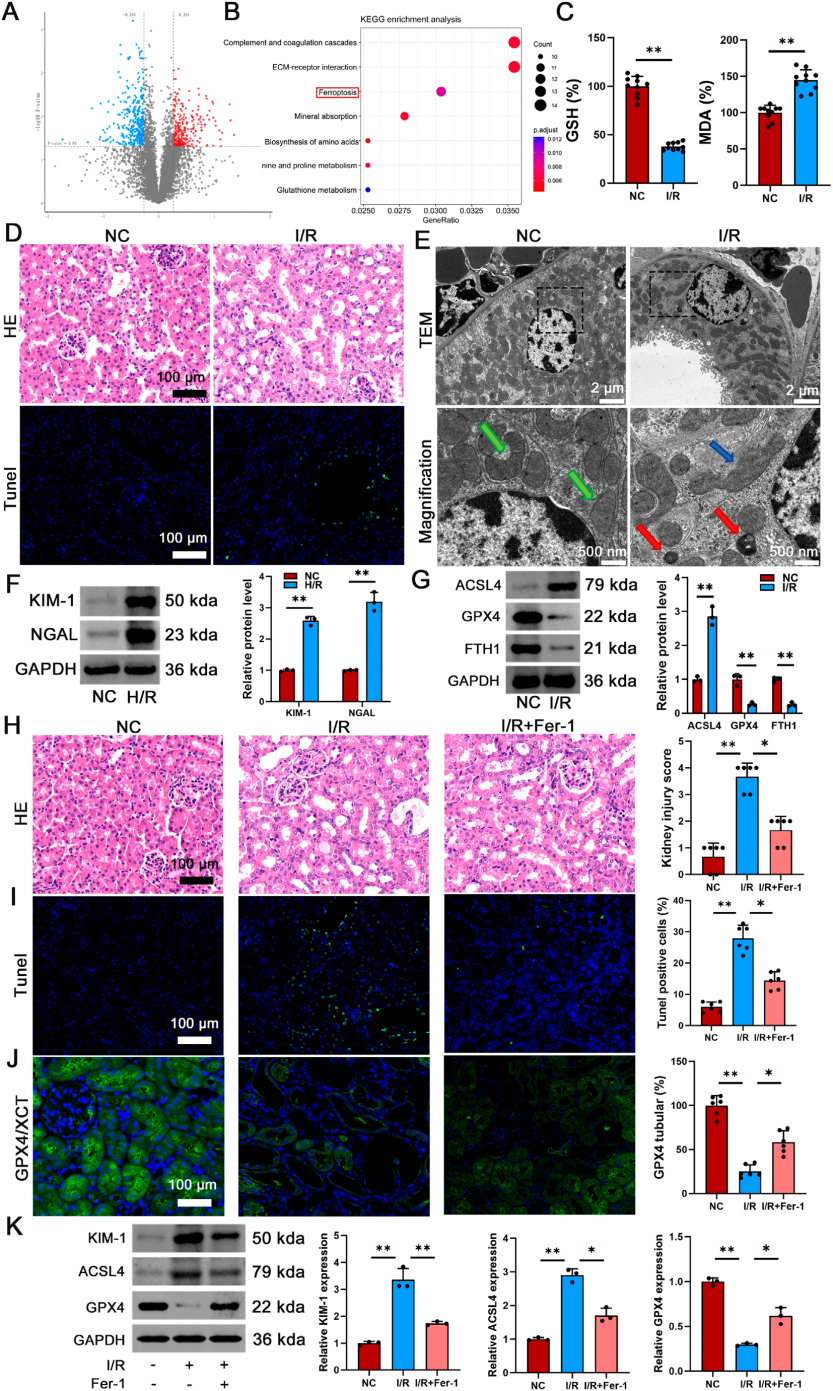
I/R‐induced AKI enhances ferroptosis. C57BL/6 mice were subjected to 30 min of ischaemia followed by 0/4/8/24 h reperfusion, with sham‐operated mice as controls. (A) Volcano plots showing the −log_10_ *p*‐value of all differentially expressed genes in NC and I/R groups, significantly up‐regulated in red and down‐regulated in blue. (B) KEGG pathway enrichment analysis reveals ferroptosis as a top altered pathway. (C) Relative values of GSH and MDA were measured, *n* = 10. (D) Representative HE staining and TUNEL immunofluorescence, scale bar = 100 μm, magnification 40×. (E) Transmission electron microscopy of mitochondrial ultrastructure: Low‐magnification view (2500×) showing mitochondrial distribution and morphology, scale bar = 2 μm; High‐magnification view (10,000×) highlighting ultrastructural details, scale bar = 500 nm. Mitochondria are pseudocoloured as follows: Green (normal morphology with intact cristae), blue (damaged mitochondria with cristae disruption) and red (ferroptotic mitochondria exhibiting characteristic shrinkage and membrane rupture). (F) Western blot assessing the expression of KIM‐1 and NGAL in NC and I/R groups, *n* = 3. (G) Western blot assessing the expression of ferroptosis related molecules in NC and I/R groups, *n* = 3. (H–J) Representative HE, TUNEL staining and GPX4 staining in kidney tissues micrographs, scale bar = 100 μm, magnification 40×. (K) Western blot analysis to assess the expression of KIM‐1, ACSL4 and GPX4 in NC group, I/R group and I/*R* + Fer‐1 group. *p < 0.05, **p < 0.01.

Proteomic profiling revealed significant ferroptosis activation during renal I/R injury. Label‐free quantitative proteomics identified 302 upregulated and 217 downregulated proteins in I/R‐treated renal tubular epithelial cells compared to normal controls (NC) (Figure 1A). KEGG pathway analysis highlighted ferroptosis as a top‐altered pathway (Figure 1B). In addition, the I/R group showed a significant decrease in GSH and an increase in MDA levels (Figure 1C). Initial findings in the AKI model induced by I/R demonstrated that the control group had intact renal tubular architecture with preserved basement membrane, clearly defined cellular structure and absence of inflammatory infiltrates. Conversely, the I/R group exhibited characteristic tubular injury patterns including lumen dilation, pronounced inflammatory cell infiltration and epithelial cell atrophy/loss. TUNEL staining showed a notable rise in apoptosis rates of renal tubular epithelial cells in the I/R group (Figure 1D). Transmission electron microscopy (TEM) further revealed well‐organised mitochondrial arrays in controls, featuring uniform morphology and tightly packed cristae, whereas I/R group mitochondria exhibited cristae fragmentation and matrix vacuolisation (Figure 1E).

The levels of kidney injury molecule‐1 (KIM‐1) and neutrophil gelatinase‐associated lipid transport protein (NGAL) were analysed to evaluate ferroptosis‐induced damage in renal tubules following I/R. The increased levels of KIM‐1 and NGAL suggested a reaction to renal damage, signifying tubular injury. Western blot analysis of KIM‐1 and NGAL protein levels in mouse kidney tissues showed a notable increase following I/R injury (Figure 1F). Western blot analysis showed a significant decrease in GPX4 and FTH1 protein levels, as well as a notable increase in ACSL4 protein levels (Figure 1G).

To mechanistically interrogate ferroptosis involvement, we administered the specific inhibitor Fer‐1. Histological analysis with haematoxylin and eosin staining revealed severe tubular injury in the I/R group, characterised by epithelial swelling, luminal cast formation and focal haemorrhage (Figure 1H). Fer‐1 treatment attenuated the worsening of morphological changes in the renal tubules, leading to a much lower renal tubular pathological injury score. The results of TUNEL staining analysis indicated a significant increase in apoptosis of the tubular epithelium in the I/R group, a phenomenon that was mitigated by Fer‐1 intervention (Figure 1I). Fer‐1 treatment improved the decreased GPX4 expression in the I/R group (Figure 1J). Additionally, these data demonstrate that Fer‐1 treatment partially reversed the up‐regulation of KIM‐1 and ACSL4, as well as the down‐regulation of GPX4 (Figure 1K). Overall, Fer‐1 showed the capability to reduce all identified alterations in the setting of I/R injury, with Ferroptosis being promoted by I/R‐induced AKI in vivo.

### 3.2 Increased Levels of HIF‐1α Were Observed in the I/R Group

**FIGURE 2:**
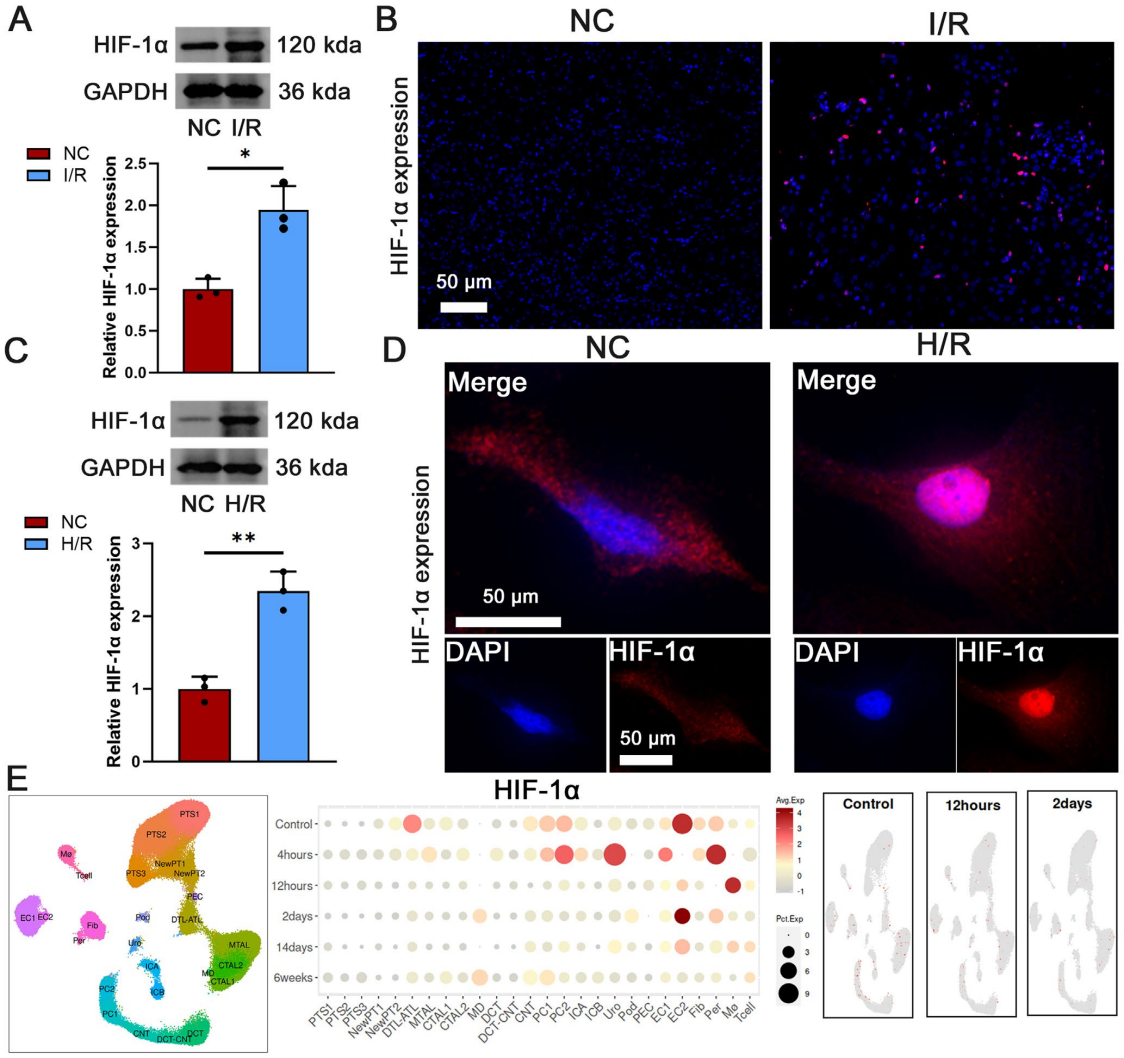
Elevated HIF‐1α expression in I/R group. (A) Western blot assessment of HIF‐1α expression in NC and I/R groups, *n* = 3. (B) Immunofluorescence photographs showing HIF‐1α in renal tissues of the I/R group, scale bar = 50 μm, magnification 100×. (C) Western blot assessment of HIF‐1α expression in NC and H/R groups, *n* = 3. (D) Immunofluorescence photographs showing HIF‐1α in renal tissues of the H/R group, scale bar = 50 μm, magnification 200×. (E) Analysis of HIF‐1α expression using a single cell database. *p < 0.05, **p < 0.01.

Hypoxia‐inducible HIF‐1α accumulation was confirmed in I/R models. Western blot and immunofluorescence demonstrated significantly increased HIF‐1α expression in the I/R group versus the non‐treated NC group (Figure 2A,B). Consistently, hypoxia‐reoxygenation (H/R) in vitro models showed a marked increase in HIF‐1α protein expression compared to the N/C group (Figure 2C,D). Subsequent single‐cell sequencing analysis (humphreyslab.com/SingleCell) of cell profiles in the NC and I/R groups identified 26 distinct cell clusters with diverse staining and labelling patterns (Figure 2E). These findings indicate that prolonged I/R time resulted in increased HIF‐1α expression across various cell clusters.

### 3.3 HIF‐1α Plays a Protective Role in AKI Induced by I/R

**FIGURE 3:**
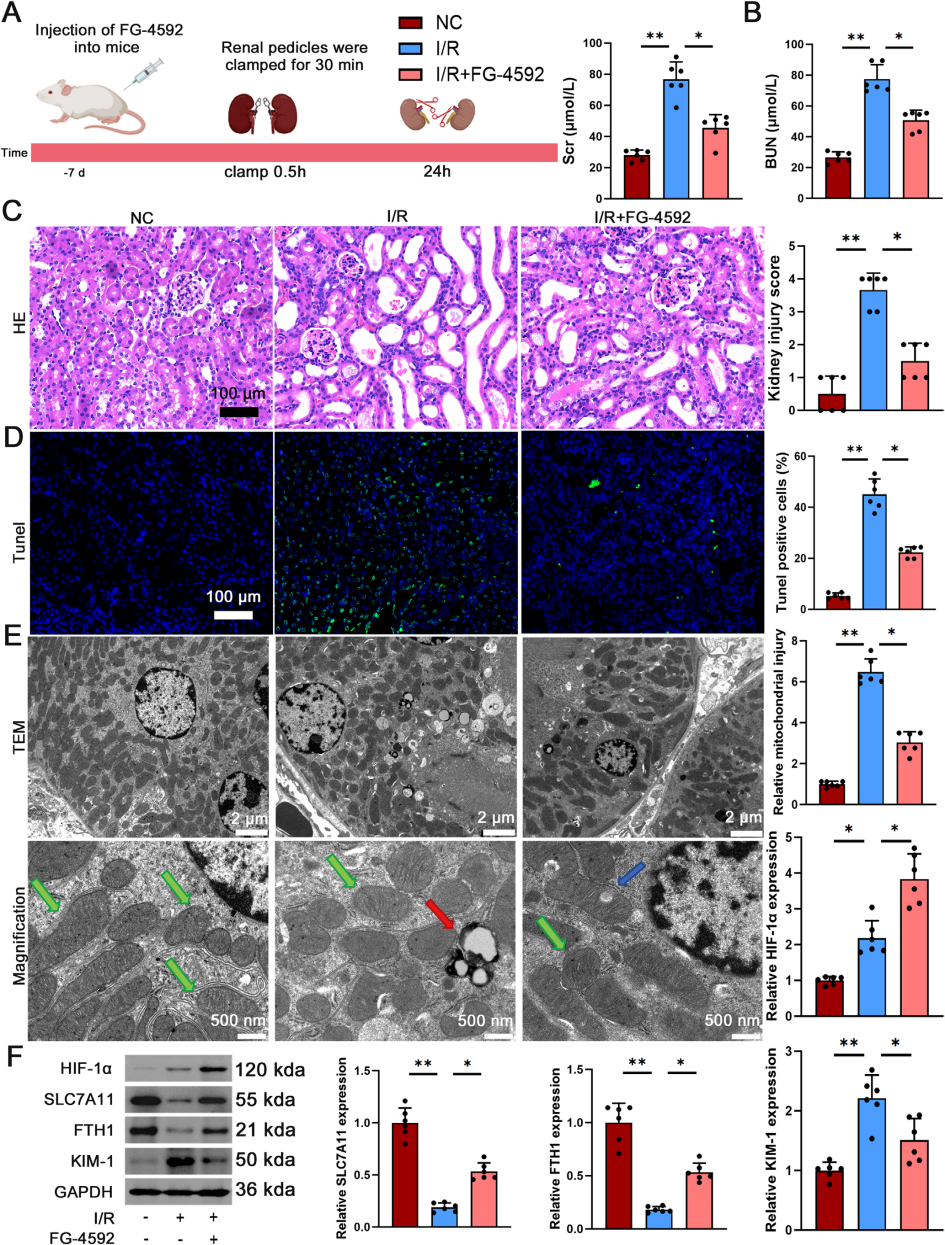
HIF‐1α plays a protective role in AKI induced by I/R. (A) Timeline protocol for establishing the I/R model. C57BL/6 mice were subjected to 30 min of renal ischaemia (clapping bilateral renal pedicles) followed by reperfusion after 24 h, with sham‐operated mice as controls. (B) Serum creatinine and Urea nitrogen levels in NC, I/R and I/*R* + FG‐4592 groups, *n* = 6. (C, D) Representative micrographs of HE, TUNEL staining in renal tissues, scale bar = 100 μm, magnification 40×. (E) Transmission electron microscopy of mitochondrial ultrastructure: Low‐magnification view (2500×) showing mitochondrial distribution and morphology, scale bar = 2 μm; High‐magnification view (10,000×) highlighting ultrastructural details, scale bar = 500 nm. Mitochondria are pseudocoloured as follows: Green (normal morphology with intact cristae), blue (damaged mitochondria with cristae disruption) and red (ferroptotic mitochondria exhibiting characteristic shrinkage and membrane rupture). (F) Western blot analysis to assess the expression of HIF‐1α, SLC7A11, FTH1 and KIM‐1 in the NC, I/R and I/*R* + FG‐4592 groups, *n* = 6. *p < 0.05, **p < 0.01.

HIF‐1α exhibits a protective role in ameliorating AKI induced by I/R. To investigate the contribution of HIF‐1α in the resistance to ferroptosis triggered by I/R, we augmented HIF‐1α expression in HK2 cells by employing the HIF prolyl hydroxylase inhibitor roxadustat (FG‐4592) (Figure 3A). Compared to the I/R group, levels of creatinine and urea nitrogen in mice treated with FG‐4592 were significantly reduced, indicating a protective effect of HIF‐1α (Figure 3B). The histological analysis of haematoxylin and eosin staining indicated that renal tubules in the normoxic NC group demonstrated a tightly aligned structure with well‐defined basement membranes, absence of edema and no inflammatory cell infiltration. In contrast, renal tubules in the I/R group exhibited significant expansion and infiltration of inflammatory cells. The extent of pathological harm was notably lessened in the I/R group that received FG‐4592 compared to the untreated I/R group, as illustrated in Figure 3C. Additionally, TUNEL staining revealed a marked increase in apoptosis levels in the I/R group, which was alleviated with FG‐4592 treatment (Figure 3D). Furthermore, examination with transmission electron microscopy showed a decrease in the size of mitochondria and an increase in the density of mitochondrial membranes in the I/R group, changes that were successfully reversed in the I/R group treated with FG‐4592 (Figure 3E). The morphological changes were significantly influenced by the administration of FG‐4592. Analysis from Figure 3F showed higher protein levels of HIF‐1α and KIM‐1 in the I/R group, with decreased levels of FTH1 and SLC7A11. Treatment with FG‐4592 in the I/R group resulted in increased expression of HIF‐1α, FTH1 and SLC7A11, along with decreased expression of KIM‐1. These results indicate that HIF‐1α appears to exert renal protection by suppressing ferroptosis during I/R‐induced AKI.

### 3.4 HIF‐1α Prevents the Onset of Apoptosis and Ferroptosis

**FIGURE 4:**
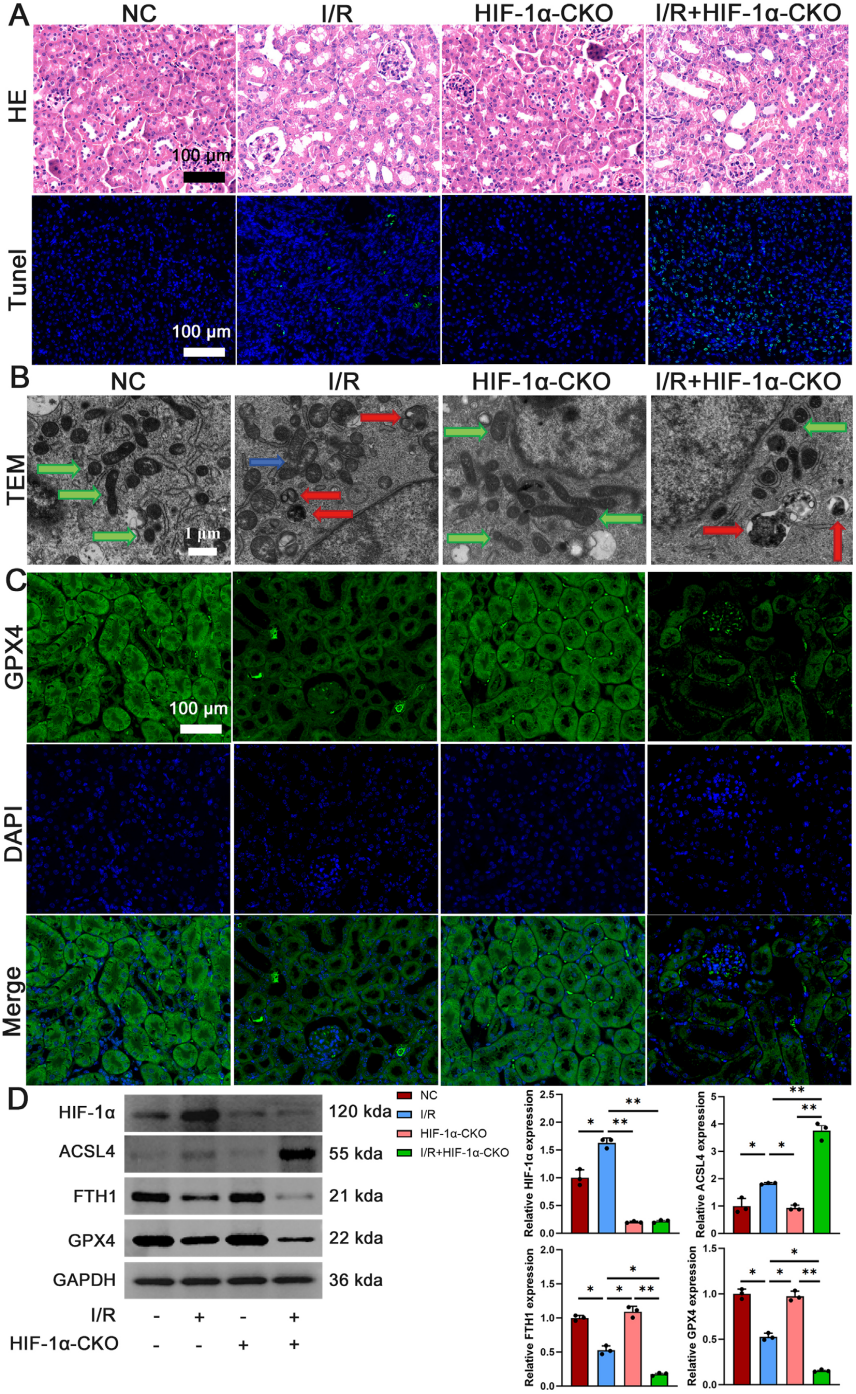
HIF‐1α prevents the onset of apoptosis and ferroptosis. (A) Representative HE staining and TUNEL immunofluorescence, scale bar = 100 μm, magnification 40×. (B) Transmission electron microscopy of mitochondrial ultrastructure: Magnification = 5000×, scale bar = 1 μm. Mitochondria are pseudocoloured as follows: Green (normal morphology with intact cristae), blue (damaged mitochondria with cristae disruption) and red (ferroptotic mitochondria exhibiting characteristic shrinkage and membrane rupture). (C) Representative micrographs GPX4 staining in renal tissues, scale bar = 100 μm, magnification 40×. (D) Western blot analysis was performed to assess the expression of HIF‐1α, ACSL4, FTH1 and GPX4 in NC group, I/R group, HIF‐1α‐CKO group and I/*R* + HIF‐1α‐CKO group, *n* = 3. *p < 0.05, **p < 0.01.

Following this, a comprehensive investigation was conducted to assess the potential protective effects of HIF‐1α against ferroptosis induced by I/R injury. Figure 4A shows the typical appearance of renal tubular epithelial cells in the control group, while the I/R group exhibited clear renal tissue damage. Notably, conditional knockout of HIF‐1α in the I/R injury (I/*R* + HIF‐1α‐CKO) group resulted in exacerbated pathological alterations compared to the I/R group, including pronounced cellular swelling, dilated lumens, complete brush border loss, disrupted tubular architecture, interstitial stenosis and marked inflammatory infiltration (Figure 4A). TUNEL staining revealed that while apoptosis rates were significantly elevated in the I/R group relative to controls, the I/*R* + HIF‐1α‐CKO group showed a significant increase in apoptotic cells compared to the I/R group. TEM ultrastructural analysis demonstrated that mitochondrial damage progression was significantly accelerated in I/*R* + HIF‐1α‐CKO compared to the I/R group, transitioning from cristae disorganisation and membrane densification in the I/R group to complete cristae fragmentation with intra‐mitochondrial vesiculation in the knockout model (Figure 4B). Moreover, GPX4 immunofluorescence intensity was significantly reduced in the I/R group compared to the normal NC group, and a further decline in GPX4 expression was seen in the I/*R* + HIF‐1α‐CKO group (Figure 4C). Western blot analysis revealed upregulation of HIF‐1α and ACSL4, alongside downregulation of FTH1 and GPX4 in the I/R group compared to NC (Figure 4D). Moreover, this observed pattern was more prominent in the I/*R* + HIF‐1α‐CKO group, collectively indicating HIF‐1α exerts protective effects against I/R‐induced apoptosis and ferroptosis.

### 3.5 HIF‐1α Knockout Aggravates Ferroptosis

**FIGURE 5:**
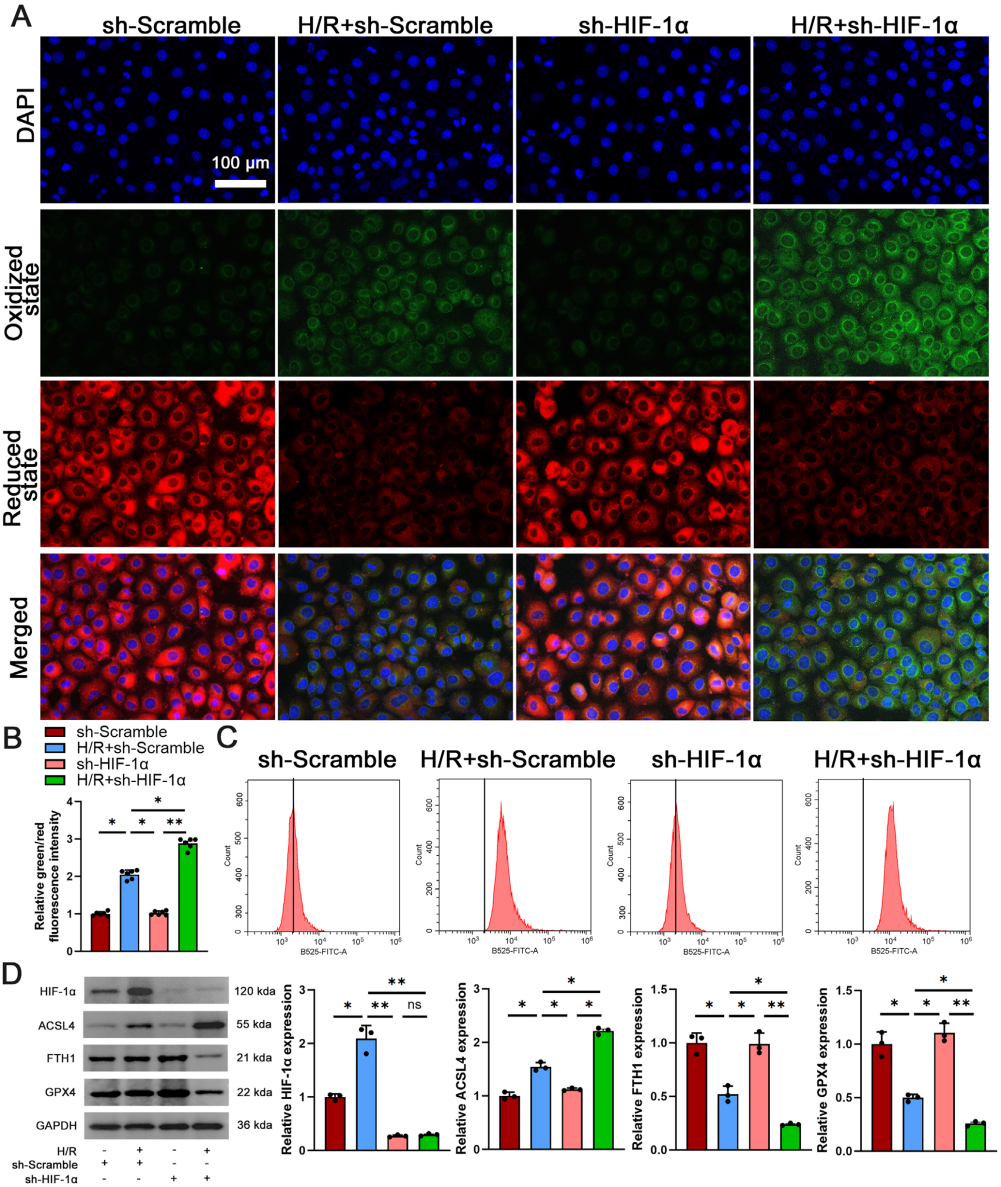
HIF‐1α knockout aggravates ferroptosis. (A) Immunofluorescence analysis of lipid peroxidation markers in sh‐Scramble, H/*R* + Scramble, sh‐HIF‐1α and H/*R* + sh‐HIF‐1α groups, *n* = 6, scale bar = 100 μm, magnification 40×. (B, C) Flow cytometry analysis of ROS levels. (D) Western blot analysis evaluated HIF‐1α, ACSL4, GPX4 and FTH1 expression in sh‐Scramble, H/*R* + Scramble, sh‐HIF‐1α and H/*R* + sh‐HIF‐1α groups, *n* = 3. *p < 0.05, **p < 0.01.

Subsequently, an evaluation of the main characteristics of ferroptosis was conducted, which included an analysis of lipid peroxidation markers and ferroptosis markers. The results depicted in Figure 5A showed a significant elevation in lipid peroxidation levels in the H/*R* + sh‐Scramble group. Reduced levels of HIF‐1α in the H/R group led to increased lipid peroxidation in cells, worsening damage and advancing ferroptosis. Flow cytometry analysis revealed higher levels of ROS in the H/*R* + sh‐Scramble group, which was worsened by HIF‐1α knockdown (Figure 5B,C). Additionally, there was an increase in HIF‐1α and ACSL4 protein expression in the H/*R* + Scramble group, along with a decrease in FTH1 and GPX4 protein expression. Furthermore, depletion of HIF‐1α in H/R‐treated cells resulted in further reduction of FTH1 and GPX4 protein levels (Figure 5D). The results indicate that HIF‐1α is involved in the process of ferroptosis and serves as a protective element in H/R‐induced AKI.

### 3.6 HIF‐1α‐Mediated I/R‐Induced AKI Is Dependent on the SLC7A11/GPX4 Signalling Pathway

**FIGURE 6:**
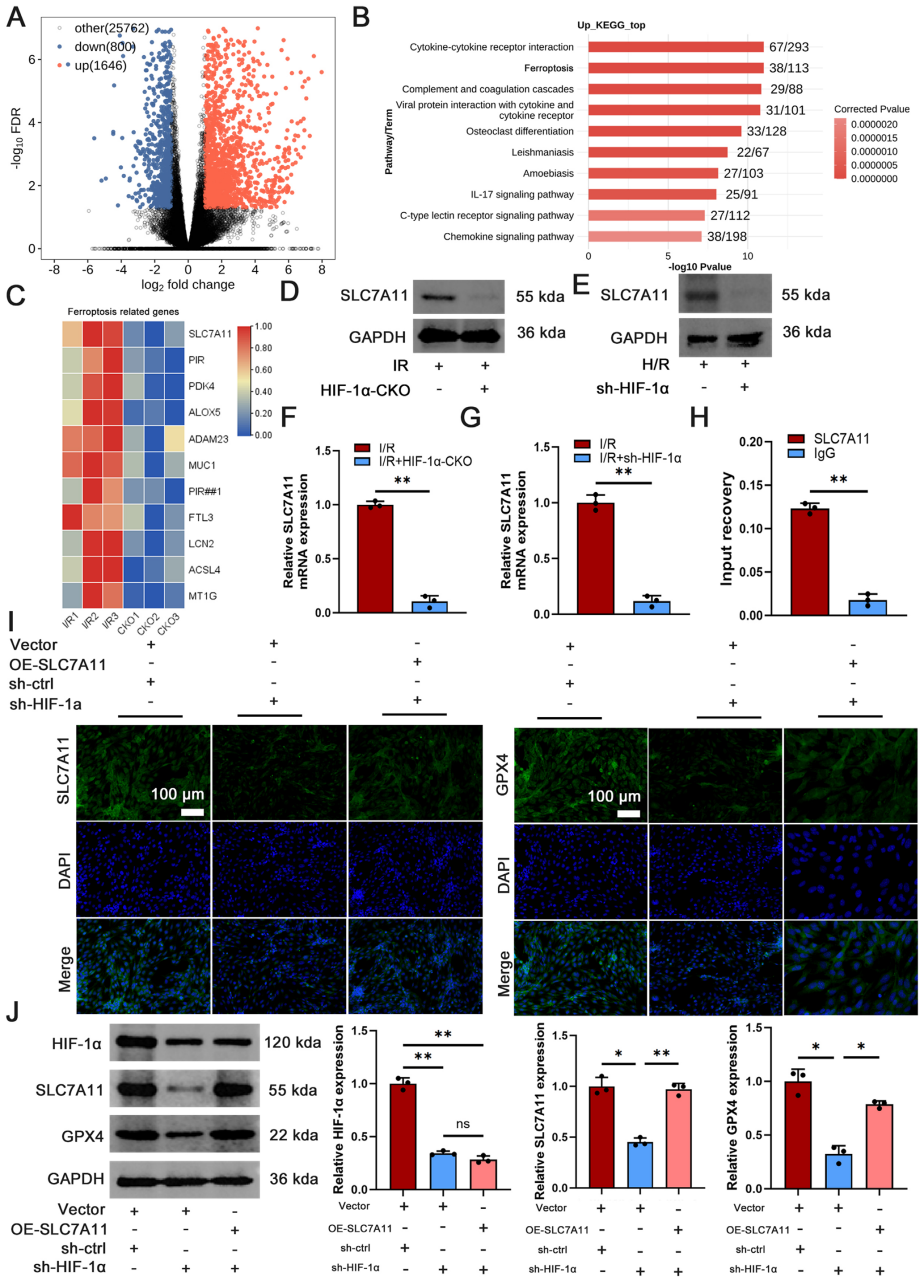
HIF‐1α‐mediated I/R‐induced AKI is dependent on the SLC7A11/GPX4 signalling pathway. (A) Volcano plot showing the −log_10_ FDR of all differentially expressed genes in the I/*R* + HIF‐1α‐CKO and I/R groups, significantly up‐regulated in red and down‐regulated in blue. (B) KEGG signalling pathway enrichment analysis of the I/*R* + HIF‐1α‐CKO and I/R groups. (C) Significantly differentiated genes of I/*R* + HIF‐1α‐CKO based on KEGG analysis. (D‐G) The mRNA and protein expression of GPX4 in I/R and I/*R* + HIF‐1α‐CKO groups, H/R and H/*R* + sh‐HIF‐1α groups were evaluated by RT‐PCR and Western blot, respectively, *n* = 3. (H) Co‐IP analysis of HIF‐1α and SLC7A11 interaction in HK2 cells under I/R conditions, *n* = 3. (I) Representative microscopic images of SLC7A11 and GPX4 in HK2 cells, scale bar = 100 μm, magnification 40×. (J) HIF‐1α, SLC7A11 and GPX4 were assessed by Western blot analysis in three different groups of HK2 cells (Vector+sh‐ctrl, Vector+sh‐HIF‐1α, OE‐SLC7A11+ sh‐HIF‐1α) expression, *n* = 3. *p < 0.05, **p < 0.01.

Proteomic analyses were conducted on both the I/R group and the I/*R* + HIF‐1α‐CKO group to gain a deeper insight into the molecular mechanisms of ferroptosis in renal I/R injury (Figure 6A–C). A volcano plot was constructed based on the proteomics data, revealing 1646 up‐regulated and 800 down‐regulated genes. Furthermore, the analysis of differential protein expression was conducted through KEGG pathway enrichment analysis, identifying ferroptosis as a pathway significantly impacted. Bubble plots displayed notable differences in gene expression between the I/R and I/*R* + HIF‐1α‐CKO groups, with the ferroptosis‐associated gene SLC7A11 exhibiting the most prominent variation. Western blot and RT‐PCR analyses supported this discovery, verifying the variations in SLC7A11 protein and mRNA levels between the two groups. The I/*R* + HIF‐1α‐CKO group showed notably reduced expression levels compared to the I/R group (Figure 6D–G). These findings aligned with the H/R model observations. Furthermore, chromatin immunoprecipitation analysis confirmed that HIF‐1α could directly influence SLC7A11 expression (Figure 6H).

Immunofluorescence was used to assess the levels of expression of the important genes SLC7A11 and GPX4 associated with ferroptosis (Figure 6I). The findings showed a notable decrease in the immunofluorescence intensity of SLC7A11 and GPX4 in the sh‐HIF‐1α group when compared to the control group. Additionally, the overexpression of SLC7A11 led to a notable increase in the fluorescence intensity of GPX4, consistent with the changes observed in Western blot protein analysis (Figure 6I,J).

### 3.7 Overexpression of iASPP Inhibits Ferroptosis

**FIGURE 7:**
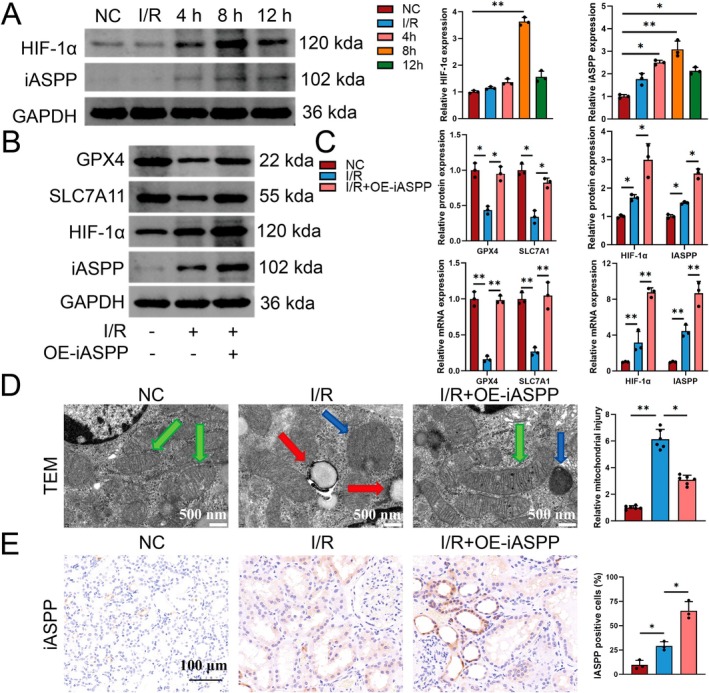
Overexpression of iASPP inhibits ferroptosis. (A) Western blot analysis of iASPP and HIF‐1α protein expression levels in HK2 cells after I/R treatment, *n* = 3. (B, C) Western blot analysis of GPX4, SLC7A11, iASPP and HIF‐1α protein expression in NC, I/R and I/*R* + OE‐iASPP groups, *n* = 3. (D) Transmission electron microscopy of mitochondrial ultrastructure: Magnification = 5000×, scale bar = 1 μm. Mitochondria are pseudocoloured as follows: Green (normal morphology with intact cristae), blue (damaged mitochondria with cristae disruption) and red (ferroptotic mitochondria exhibiting characteristic shrinkage and membrane rupture). (E) Representative immunohistochemical analysis of iASPP expression in NC, I/R and I/*R* + OE‐iASPP groups, scale bar = 100 μm, magnification 40×. *p < 0.05, **p < 0.01.

Figure 7A demonstrates a progressive rise in HIF‐1α and iASPP protein levels during the I/R process, reaching maximal expression at 8 h post‐I/R followed by gradual attenuation. Western blot and RT‐PCR analyses were utilised to evaluate the expression levels of ferroptosis‐related proteins (Figure 7B,C). Overexpression of iASPP led to increased levels of GPX4, SLC7A11 and HIF‐1α proteins and mRNA compared to the I/R group, reversing the GPX4 and SLC7A11 decrease seen during I/R. Analysis using TEM showed a decrease in mitochondrial size and an increase in mitochondrial membrane integrity in the I/R group, but significant improvement in mitochondrial damage was observed after iASPP overexpression (Figure 7D). Immunohistochemistry was used to assess the expression of iASPP in the tissues, showing notably higher levels in the I/R group in comparison to the NC group. Moreover, overexpression of iASPP led to a further augmentation of iASPP expression (Figure 7E).

**FIGURE 8:**
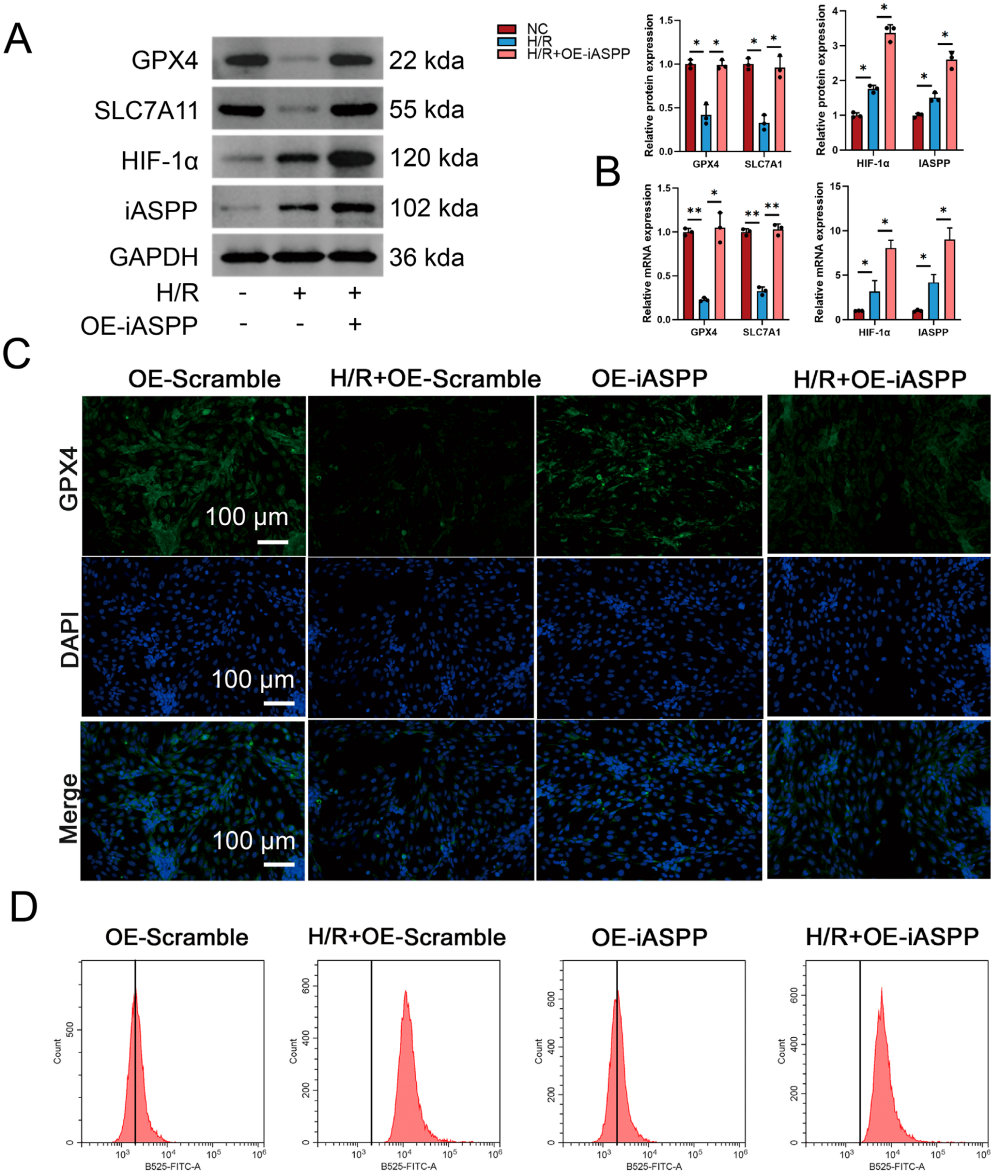
Overexpression of iASPP inhibits ferroptosis. (A, B) Western blot and RT‐PCR were used to analyse the expressions of ferroptosis‐related proteins GPX4, SLC7A11, iASPP and HIF‐1α in NC, H/R and H/*R* + OE‐iASPP groups, *n* = 3. (C)The expression of GPX4 in OE‐Scramble, H/*R* + OE‐Scramble, OE‐iASPP and H/*R* + OE‐iASPP groups was analysed by immunofluorescence method, scale bar = 100 μm, magnification 40×. (D) ROS levels in OE‐Scramble, H/*R* + OE‐Scramble, OE‐Scramble, H/*R* + OE‐iASPP were analysed by flow cytometry. *p < 0.05, **p < 0.01.

Similarly, the H/R model showed increased levels of GPX4, SLC7A11 and HIF‐1α protein and mRNA expression due to the upregulation of iASPP, as demonstrated by Western blot and RT‐PCR analyses when compared to the H/R group (Figure 8A,B). Immunofluorescence was used to evaluate the levels of GPX4, showing a notable reduction in the H/*R* + OE‐Scramble group compared to the OE‐Scramble group. Additionally, GPX4 expression in the H/R group decreased even more after iASPP overexpression treatment compared to the H/*R* + OE‐Scramble group (Figure 8C). Flow cytometry analysis revealed a significant increase in ROS levels in the H/*R* + OE‐Scramble group, which then decreased with iASPP overexpression in the H/R group (Figure 8D). These results collectively suggest that iASPP‐mediated ferroptosis functions as a vital protective mechanism in I/R‐induced AKI.

### 3.8 Inhibition of iASPP Expression Can Promote Ferroptosis

**FIGURE 9:**
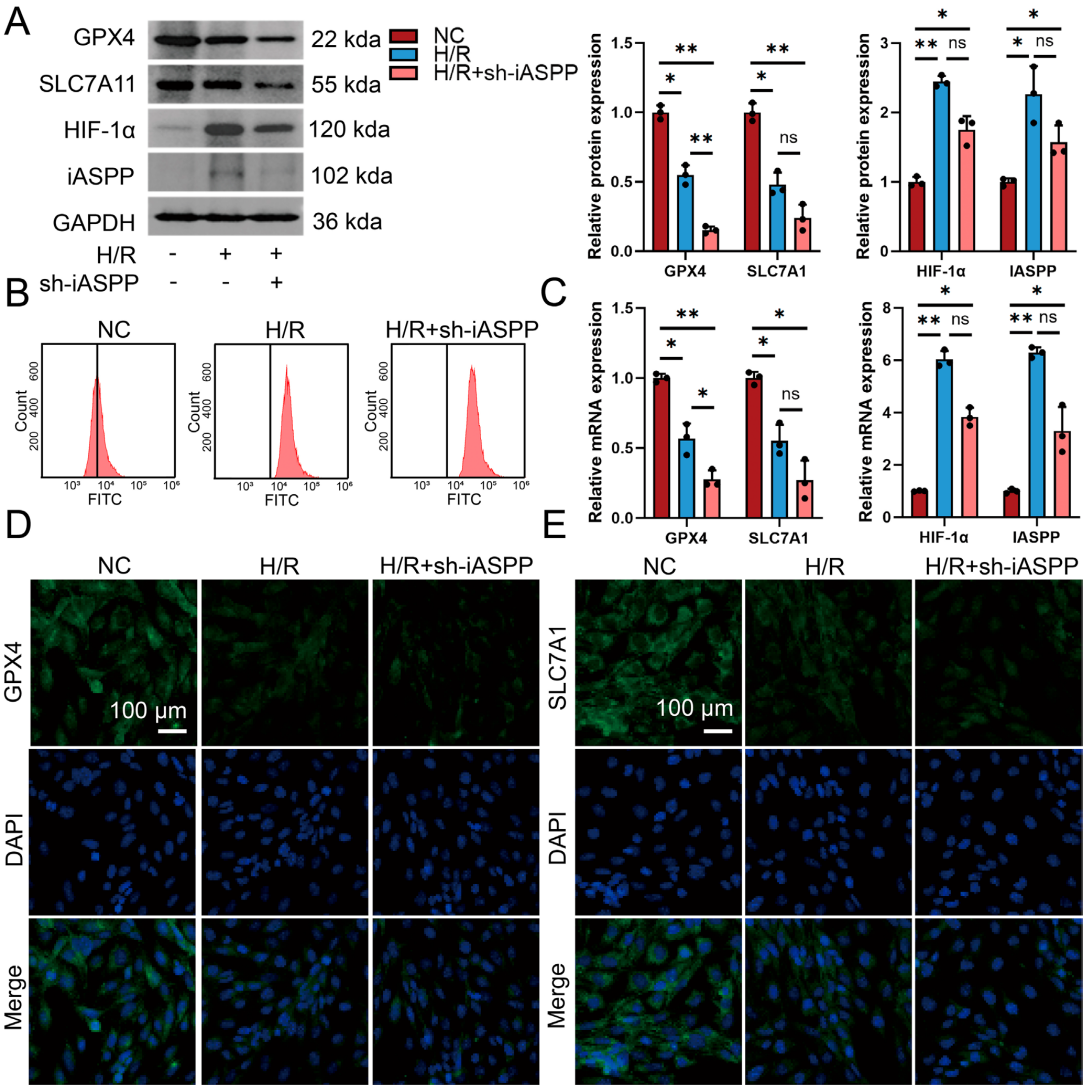
Inhibition of iASPP expression can promote ferroptosis. (A) Western blot analysis of ferroptosis‐related proteins GPX4, SLC7A11, iASPP and HIF‐1α in NC, H/R and H/*R* + sh‐iASPP groups, *n* = 3. (B) Flow cytometry analysis of NC, H/R and H/*R* + ROS levels in the sh‐iASPP group. (C) The expressions of ferroptosis‐related proteins GPX4, SLC7A11, iASPP and HIF‐1α in NC, H/R and H/*R* + sh‐iASPP groups were analysed by RT‐PCR, *n* = 3. (D, E) The expressions of GPX4 and SLC7A11 in NC, H/R and H/*R* + sh‐iASPP were analysed by immunofluorescence method, scale bar = 100 μm, magnification 40×. *p < 0.05, **p < 0.01.

We conducted a detailed analysis of iASPP knockout expression in HK2 cells following H/R. The results indicated a reduction in the protein levels of GPX4 and SLC7A11 following H/R, concurrent with upregulation of HIF‐1α and iASPP (Figure 9A). Additionally, the expressions of GPX4 and SLC7A11 were further diminished subsequent to iASPP knockout. After iASPP knockout, flow cytometry analysis showed an increase in ROS levels in the H/R group, with a further escalation seen post iASPP knockout (Figure 9B). RT‐PCR analysis showed that after iASPP deletion, GPX4 and SLC7A11 mRNA levels were notably decreased compared to H/R conditions, with HIF‐1α mRNA levels also lower than those in the H/R group (Figure 9C). Immunofluorescence results showed that the fluorescence intensity of GPX4 and SLC7A11 increased in the H/R group, but decreased when iASPP was removed (Figure 9D).

## 4 Discussion

The study investigated the therapeutic potential of iASPP and HIF‐1α in relation to ferroptosis and their combined effects on AKI induced by I/R in both laboratory and animal studies. The findings showed: (1) I/R‐induced AKI enhances ferroptosis; (2) Elevated HIF‐1α expression in the I/R group; (3) HIF‐1α antagonises ferroptosis through SLC7A11; (4) HIF‐1α prevents the onset of apoptosis and ferroptosis; (5) HIF‐1α knockout aggravates ferroptosis; (6) Overexpression of iASPP inhibits ferroptosis; (7) Inhibition of iASPP expression can promote ferroptosis; (8) HIF‐1α‐mediated I/R‐induced AKI is dependent on the SLC7A11/GPX4 signalling pathway.

Studies have shown that ferroptosis, a distinct iron‐dependent form of regulated cell death mechanistically independent of apoptosis, involves iron‐dependent lipid peroxidation and iron buildup during cell death [20, 21, 22]. It is unclear how ferroptosis occurs, but recent research suggests that it may play a significant role in neurodegeneration, inflammation and intrarenal injury [23, 24, 25]. Prior studies have posited that apoptosis is pivotal in the initial pathophysiology of AKI, and as research progresses, ferroptosis and necrotic apoptosis have emerged as central factors in I/R‐induced AKI [26]. This research explores the role of ferroptosis in exacerbating AKI caused by I/R. Recent findings have shown a growing connection between ferroptosis and the development of I/R‐induced injury in different organs. Blocking ferroptosis has shown effectiveness in improving organ damage caused by I/R.

iASPP, a crucial controller of cell death, interacts with p53 and influences its function within the nucleus. Investigation into the potential involvement of iASPP in AKI is warranted. The expanding repertoire of proteins interacting with iASPP suggests its pivotal role in diverse biological processes [27, 28]. According to our results, iASPP suppresses ferroptosis and ameliorates I/R‐induced AKI, proving its protective properties in a variety of I/R‐induced injury models. iASPP suppresses ferroptosis, reducing lung injury caused by intestinal I/R, while hypothermia reduces brain injury and apoptosis induced by I/R by increasing iASPP levels in mice [18, 29]. Furthermore, Ge et al. [30] have observed that iASPP can diminish ROS levels, mitigate DNA damage and impede cell apoptosis by activating Nrf2‐mediated anti‐ROS mechanisms.

Prior research has demonstrated that iASPP facilitates unique aspects of angiogenesis and glycolysis in a manner dependent on HIF‐1α, indicating a potential significant role for iASPP through HIF‐1α [19]. We posited that iASPP and HIF‐1α‐mediated ferroptosis may offer protection against AKI resulting from lethal I/R. The assessment of ferroptosis‐related factors was conducted through the generation of HIF‐1α‐conditional knockout mice and a HIF‐1α‐knockout HK‐2 cell line in an anoxic‐reoxygenation model. In addition to exacerbated AKI caused by I/R, our research also suggested that IASPP has a protective effect against I/R‐induced AKI. The discovery highlights the crucial importance of HIF‐1α in the formation of AKI caused by I/ *R. Prior* research has also revealed the defensive role of HIF‐1α in ferroptosis. A recent investigation conducted by Liu et al. showed that Sirtuin4 can alleviate intense acute pancreatitis by adjusting HIF‐1α/HO‐1‐mediated ferroptosis [30, 31]. Yang et al. [32] demonstrated that HIF‐1α boosts the generation of lactic acid and triggers SLC1A1 to inhibit ferroptosis in solid tumours. Furthermore, Ling et al. discovered that hypoxia‐induced LncRNA‐PMAN could hinder ferroptosis in gastric cancer with peritoneal metastases by promoting ELAVL1 cytoplasmic translocation [33].

HIF‐1α can be found in all mammalian tissue cells, especially in renal tubular epithelial cells. Fu et al. [34] demonstrated that the HIF‐1α‐BNIP3 pathway is essential in decreasing ROS generation in I/R injury by controlling the cell division of renal tubule cells, thus alleviating kidney harm. Rosallistat (FG‐4592), a prolyl hydroxylase inhibitor, effectively blocks prolyl hydroxylase activity by acting like ketoglutaric acid, thus stopping the breakdown of HIF‐1α. Studies have demonstrated that FG‐4592 increases HIF‐1α levels, resulting in reduced production of inflammatory factors and protection against kidney injury caused by I/R [35]. Elevated fluorescence and protein concentrations of KIM‐1 and ACSL4 were detected in the I/R group, with a reduction in GPX4 expression. Treatment with Fer‐1, a ferroptosis inhibitor, resulted in decreased levels of KIM‐1 and ACSL4 expression, increased GPX4 expression and alleviation of the corresponding apoptosis inhibition.

Furthermore, our investigation also delved into the involvement of additional ferroptosis‐related molecules. Diminished fluorescence and protein expression levels of SLC7A11 and GPX4 were observed in sh‐HIF‐1α, resulting in reduced apoptosis levels. Previous research has indicated that downregulation of SLC7A11 expression can mitigate acute lung injury resulting from intestinal I/R by modulating ferroptosis [36]. Our research, combined with previous studies, suggests that iASPP could have a protective impact by increasing the levels of ferroptosis‐related proteins FTH1, SLC7A11 and GPX4 via HIF‐1α. This supports our finding that iASPP helps alleviate AKI by influencing protective mechanisms through the HIF‐1α/SLC7A11 pathway.

While emerging evidence suggests that complementary mechanisms such as the FSP1‐CoQ10 system and dihydroorotate dehydrogenase‐mediated pathways may contribute to ferroptosis modulation, the SLC7A11‐GSH‐GPX4 axis remains the central regulatory hub of this process. In the absence of SLC7A11 and GPX4 proteins, ROS accumulate, resulting in lipid peroxidation, subsequent membrane damage and cell death. The study showed that reducing HIF‐1α led to lower SLC7A11 levels, suggesting HIF‐1α directly controls SLC7A11 expression by interacting with its promoter. This suggests SLC7A11 could be a new target gene for HIF‐1α. Notably, in cerebral I/R models, HIF‐1α accumulation commenced 1 h post‐reperfusion and persisted for a minimum of 7 days following cerebral ischaemia [37, 38, 39]. This temporal sequence mirrors the CIR‐induced SLC7A11 expression and X_C_^−^function profile as reported by Hsieh CH, implying a potential role for HIF‐1α in sustaining prolonged and stable CIR‐induced SLC7A11 expression and systemic X_C_^−^function [40].

Overall, our results indicate a new mechanism of ferroptosis associated with increased iASPP expression, which inhibits ferroptosis through the HIF‐1α/SLC7A11 pathway. This regulatory mechanism attenuates I/R‐induced ferroptosis caused by I/R in both live and artificial models, ultimately preventing the progression of AKI. Our findings demonstrate that iASPP modulates iron homeostasis in AKI induced by I/R, providing possible targets for pharmacological intervention in managing this disease.

Acknowledgment of the study limitations is necessary since it did not explore the exact molecular interaction between iASPP and HIF‐1α. Therefore, further research is required to elucidate the specific mechanism involved. Various cell types, such as renal macrophages, peritubular microvascular endothelial cells and tubular epithelial cells, are crucial to evaluate due to AKI from I/R. While our study focused on tubular epithelial cells, upcoming investigations should encompass a broader range of cell types to gain a deeper understanding of the protective effects of iASPP and HIF‐1α during AKI induced by apoptosis and I/R.

## Author Contributions

**Peng Kang:** conceptualization (equal), writing – original draft (equal). **Xiangjun Zhou:** investigation (equal), writing – review and editing (equal). **Sheng Zhao:** methodology (equal), writing – review and editing (equal). **Weimin Yu:** software (equal), writing – review and editing (equal). **Zehua Ye:** validation (equal), writing – review and editing (equal). **Fan Cheng:** funding acquisition (equal), writing – review and editing (equal).

## Ethics Statement

All animal experiments were approved by the Animal Ethics Committee of the Renmin Hospital of Wuhan University (approval number: WDRM20200904).

## Consent

The authors have nothing to report.

## Conflicts of Interest

The authors declare no conflicts of interest.

## Data Availability

The datasets used and/or analyzed during the current study available from the corresponding author on reasonable request.
